# Association of oxytocin level and less severe forms of childhood maltreatment history among healthy Japanese adults involved with child care

**DOI:** 10.3389/fnbeh.2015.00138

**Published:** 2015-06-23

**Authors:** Rie Mizuki, Takeo Fujiwara

**Affiliations:** Department of Social Medicine, National Research Institute for Child Health and DevelopmentTokyo, Japan

**Keywords:** oxytocin, childhood abuse history, child abuse, child maltreatment, social stress

## Abstract

**Background:** Oxytocin (OT) is known to play a role in stress regulation. The association between childhood maltreatment history and neuropeptide OT concentration is inconsistent due to the varying degrees of severity of childhood maltreatment, among other contributing factors. Less severe forms of childhood maltreatment history might enhance OT concentrations as a response to coping with social stress within the family. The purpose of this study is to investigate the association between less severe forms of childhood maltreatment history and OT concentrations among healthy adults.

**Method:** Eighty adults (49 women and 31 men) with 18- to 48-month-old children were recruited using a snowball sample in Tokyo, Japan. Urine samples were collected for OT measurement. Less severe (low and moderate) childhood maltreatment history, including physical abuse, physical neglect, emotional abuse, emotional neglect, and sexual abuse, was assessed using the self-report questionnaire, the Childhood Trauma Questionnaire.

**Results:** Less severe physical abuse was significantly associated with higher OT concentration after adjusting for age (*p* = 0.014). Also, less severe forms of physical abuse were independently significantly associated with higher OT concentration after controlling for other types of childhood maltreatment (*p* = 0.027). A positive dose-response association between the number of less severe childhood maltreatment types and OT concentration was observed (*p* = 0.031).

**Conclusion:** A history of less severe forms of childhood physical abuse was associated with higher OT concentration in healthy adults. Poly-victimization of several types of less severe childhood maltreatment was also associated with higher OT concentrations. Less severe forms of childhood maltreatment might enhance OT concentrations in order to cope with social stress.

## Introduction

Oxytocin is a neuropeptide that plays an important role not only in social bonding but also in the regulation of stress and anxiety (Carter et al., 1992; Kormos and Gaszner, 2013; Peters et al., 2014). Stress reaction is usually formed at an early age of development in humans and animals. In rats, previous studies have reported that rats that were licked and groomed by a parent reacted better to stress, and showed an epigenetic change in the hippocampus-pituitary-adrenal (HPA) axis during infancy (Liu et al., 1997; Meaney, 2001). Further, rats that were licked and groomed by parents have also shown increased OT levels (Francis et al., 1999, 2000; Champagne et al., 2001). Similar results were also reported in humans, that is, the salivary OT concentrations of infants whose parents practiced responsive care, represented by high affect parent-infant synchrony, were higher than infants raised by parents who showed low affect synchrony (Feldman et al., 2010).

In the same context, childhood maltreatment, which can be considered as “the most visible and obvious indicator of dysfunctional parenting” (Holden, 2010), is associated with lower OT concentration in victimized children. Heim et al. (2009) reported that adult women with severe childhood maltreatment showed significantly lower OT concentrations in cerebrospinal fluid compared with women without a history of childhood maltreatment (Heim et al., 2009). Also, children who had been raised in neglectful institutional care in the first few years of life due to loss of parents had lower urinary OT concentrations (Wismer Fries et al., 2005). Furthermore, physically healthy adult men who experienced adverse life experiences from early childhood up to 13 years of age also showed lower plasma OT concentrations (Opacka-Juffry and Mohiyeddini, 2012). These findings can be interpreted as evidence that lower OT concentration might be associated with the child withdrawing from the stressor, that is, the caregiver who severely maltreats the offspring, because lower OT concentration can lead to a decrease in social behavior. However, strategies to deal with such stress for less severe forms of child maltreatment might be different, because of the role OT plays in the “tend-and-befriend” behavior of dealing with stress (Taylor et al., 2000). For example, Seltzer et al. (2014) reported that urinary OT concentrations after social stress exposure among maltreated girls were higher than the controls (Seltzer et al., 2014), suggesting that OT concentrations in maltreated girls exposed to social stress were enhanced to deal with this stress through more frequent social behaviors. Thus, we hypothesized that children who experienced less severe forms of maltreatment exhibit “tend-and-befriend” behaviors to deal with stress, including trying to get along with their caregiver or seeking help from others, with enhanced OT concentration serving as one potential underlying mechanism.

Thus, the purpose of this study is to test the hypothesis that low and moderate childhood maltreatment history increases OT levels among healthy adults.

## Materials and methods

### Participants

The study was approved by the Ethics Committee of the National Institute for Public Health, and all participants signed informed consent forms prior to enrollment in the study. Details about participant recruitment and eligibility criteria have already been reported elsewhere (Fujiwara et al., 2012). In short, 81 participants (49 women and 31 men who were spouses of female participants) were recruited for the study in Tokyo, Japan using a convenience snowball sample. Snowball sampling is regarded as a suitable method to use in studies that, like this one, focus on a sensitive issue (Biernacki and Waldorf, 1981). The eligibility criteria restricted the sample to women and men with 18–48-month-old children. All women were married, were not breastfeeding at the time of the study, and were the child's main caregiver.

### Procedure and oxytocin analysis

Procedures and oxytocin analysis methodology have previously been described in detail elsewhere (Fujiwara et al., 2012). Research coordinators visited participants' homes for approximately 1 h between 11 a.m. and 2 p.m. and sent questionnaires to participants in advance of the visit, which were collected during the home visit. We collected a 1-mL urine sample in a tube to which a 40−μl aliquot of sodium citrate buffer (0.03 M sodium citrate, 25 mM EDTA, and 0.35 mM 1,10-phenanthroline) was added, and immediately stored the samples in a cooler box at 4°C for a maximum of 2 h and then at −20°C in the laboratory.

OT concentrations in the urine samples were measured by a competitive radioimmunoassay, as described elsewhere (Sudo et al., 1978). In brief, we created rabbit antiserum specific for human OT by immunizing a rabbit four times with recombinant human OT (ASKA Pharmaceutical. Co., Ltd., Tokyo, Japan) combined with water-soluble carbodiimide (Nakarai Tesque, Tokyo, Japan). Then, we decomplimented the urine sample at 56°C for 30 min, and the supernatant was extracted after centrifugation (3000 rpm, 10 min, 4°C). We designated the decomplimented sample and the same amount of ^125^I-labeled OT (Perkin Elmer Life Sciences, Inc., Boston MA) for use in an assay tube (Shionogi, Tokyo, Japan). Then, we added rabbit anti-OT serum to each assay tube, followed by incubation for 2 days at 4°C. Next, we added goat anti-rabbit IgG serum (ASKA Pharmaceutical. Co., Ltd., Tokyo, Japan) to each assay tube, followed by incubation for 1 day at 4°C. After centrifugation, we measured the radioreactivity of the pellet by a gamma counter (Auto Well Gamma System ARC-1000M, Aloka, Tokyo Japan). The minimal detection limit of this assay was 3 μU/ml (1 μU of OT is equivalent to 1.776 pg) according to the standard curve. We performed all assays in duplicate, and the assay's intra- and inter-assay coefficients of variability were <14.2%. We standardized the concentration of OT in the urine according to the urinary creatinine concentration. We measured urinary creatinine using the alkaline picrate colorimetric method (modified Jaffe).

### Childhood maltreatment history

The Childhood Trauma Questionnaire (CTQ) is a 25-item self-report questionnaire that assesses five types of childhood maltreatment: physical neglect, emotional neglect, physical abuse, emotional abuse, and sexual abuse (Bernstein et al., 2003). The CTQ defined the severity of each maltreatment type as none, low, moderate, and severe by using corresponding cut-off scores. Low and moderate forms of childhood maltreatment used in the CTQ were defined as “less severe forms of maltreatment” in this study and were scored in a range between 8 and 12 for physical neglect, 10 and 17 for emotional neglect, 8 and 12 for physical abuse, 9 and 15 for emotional abuse, and 6 and 12 for sexual abuse, following the cut-off scores of the CTQ (Bernstein et al., 2003). Scores below these ranges were defined as no maltreatment, and scores above these ranges were defined as severe maltreatment (Bernstein et al., 2003).

One female participant reported a severe form of emotional neglect and was excluded from the analyses. Other severe forms of maltreatment were not reported. The sample was dichotomized into either having low and moderate childhood maltreatment history or no childhood maltreatment history.

### Covariates

Participants' age, sex, and mental health status were potential covariates. Mental health status was measured with the Depression Anxiety Stress Scales (DASS) (Lovibond and Lovibond, 1995a). DASS was a 42-item self-report questionnaire consisting of three subscales for depression, anxiety, and stress. Participants answered the questionnaire using a 4-point Likert scale and responses were summed up to derive a total score for each subscale ranging from 0 to 42.

### Statistical analysis

First, correlation analyses among five different types of maltreatment were conducted. Second, *t*-tests were conducted to observe the impact of sex and age on OT. Third, bivariate regression analyses were performed to examine the association of OT with low and moderate childhood maltreatment history, in which Model 1 was adjusted for age, and Model 2 was adjusted for age and five different types of maltreatment (physical neglect, emotional neglect, physical abuse, emotional abuse, and sexual abuse). Fourth, regression analysis was conducted to assess the dose-response association between the number of types of maltreatment and OT concentration.

## Results

Sample characteristics are shown in Table 1. The mean age of participants was 36.2 years old with a standard deviation (*SD*) of 3.4. Over half of participants had only one child, more than 90% reported their health as good or better, and over 80% received a level of education consistent with some college or more. The mean scores of depression, anxiety, and stress scales measured by DASS were comparable to past studies with non-clinical samples (Lovibond and Lovibond, 1995b; Muto et al., 2011).

**Table 1:** **Sample characteristics (*****n*** **= 80)**.

		***N* or Mean**	**% or SD**
Age	Year	36.2	3.4
Sex	Male	31	38.8
Number of children	1	43	53.8
	2	27	33.8
	3	10	12.5
Self-rated health	Excellent	34	42.5
	Very good	28	35.0
	Good	13	16.3
	Fair/poor	5	6.3
Education	High school or less	13	16.3
	Some college	29	36.3
	College or more	38	47.5
Mental health	Depression	2.0	4.6
	Anxiety	2.5	4.1
	Stress	5.2	5.3
Childhood maltreatment (Low and moderate, dichotomized from CTQ score)	Physical neglect	24	30.0
	Emotional neglect	32	40.0
	Physical abuse	5	6.3
	Emotional abuse	7	8.8
	Sexual abuse	4	5.0
No. of low and moderate maltreatment types experienced	0	34	42.5
	1	27	33.8
	2	13	16.3
	3+	6	7.5

*CTQ, Childhood Trauma Questionnaire*.

In this study, 37.5% of participants experienced low childhood maltreatment and 20% experienced moderate childhood maltreatment, where both low and moderate were defined as less severe forms of childhood maltreatment. Of these participants, 30.0% reported a history of less severe forms of physical neglect, 40.0% reported less severe forms of emotional neglect, 6.3% reported less severe forms of physical abuse, 8.8% reported less severe forms of emotional abuse, and 5.0% reported less severe forms of sexual abuse. In terms of the number of less severe forms of childhood maltreatment types experienced, 16.3% reported two types of maltreatment, and 7.5% reported three or more types of maltreatment.

Pearson's correlation analysis between each type of less severe form of childhood maltreatment indicated that physical neglect was significantly positively correlated with emotional neglect (*r* = 0.29, *p* < 0.05) (Table 2). Further, emotional neglect was significantly positively correlated with emotional abuse (*r* = 0.32, *p* < 0.05), and physical abuse was significantly positively correlated with emotional abuse (*r* = 0.26, *p* < 0.05). Other types of childhood maltreatment were not significantly correlated. Despite statistical significance, the values of these correlation coefficients between each type of less severe form of childhood maltreatment indicated that the correlations were weak.

**Table 2:** **Correlation matrix for less severe forms of maltreatment types**.

**Total**	**Physical neglect**	**Emotional neglect**	**Physical abuse**	**Emotional abuse**
Physical neglect	−			
Emotional neglect	**0.25**	−		
Physical abuse	−0.06	0.11	−	
Emotional abuse	0.09	**0.29**	**0.29**	−
Sexual abuse	−0.03	0.16	−0.06	0.13

*Significant coefficients at p < 0.05 are in bold*.

The association between OT and demographics is presented in Table 3. Men had a slightly higher OT concentration (mean = 112.8; SD = 42.1) compared with women (mean = 107.5; *SD* = 38.2), although the difference was not statistically significant. The mean OT concentration of the younger group (less than 36 years old) was lower (mean = 99.6; *SD* = 36.6) than the older group (mean = 116.5; *SD* = 39.6) at a marginal trend level (*p* = 0.057). The association between mental health status and OT was found not to be significant in this sample. Thus, only age was included as a covariate in subsequent analyses.

**Table 3:** **Oxytocin concentration by sex and age group**.

		***n***	**Oxytocin concentration (μU/ml per creatinine g/L)**	***SD***	***t*-value**	***p*-value**
Total		80	109.6	39.1	−	−
Sex	Female	49	107.5	38.2	0.59	0.555
	Male	31	112.8	42.1		
Age	≤35	33	99.6	36.6	1.93	0.057
	36+	47	116.5	39.6		

The association between less severe forms of childhood maltreatment and urinary OT concentration is shown in Table 4. Results of the bivariate regression analyses indicated that the presence of less severe forms of physical abuse history was significantly positively associated with OT concentration (coefficient = 42.97; *p* = 0.016). The presence of any other type of maltreatment history did not result in statistical significance. Also, when age was adjusted in Model 1, less severe forms of physical abuse were still significantly positively associated with OT concentration (coefficient = 43.35; *p* = 0.014). Due to correlations among five maltreatment types, we simultaneously included those types in Model 2, as well as age, in order to observe the independent impact of each maltreatment type on OT concentration. Here, only less severe forms of physical abuse significantly impacted OT concentration, after ruling out the variance accounted for by other maltreatment types (*p* = 0.027).

**Table 4:** **Regression coefficients of oxytocin by less severe forms of childhood maltreatment types**.

		**Oxytocin concentration**		**Bivariate**		**Model 1^†^**		**Model 2^††^**	
		***Mean***	***SD***	***B***	***p*-value**	***B***	***p*-value**	***B***	***p*-value**
Any maltreatment	(−)	106.17	30.73	ref.		ref.		ref.	
	(+)	112.06	44.41	5.89	0.508	5.28	0.547	—	—
Physical neglect	(−)	106.93	37.13	ref.		ref.		ref.	
	(+)	115.69	43.48	8.75	0.362	6.16	0.520	5.26	0.589
Emotional neglect	(−)	104.82	34.12	ref.		ref.		ref.	
	(+)	116.67	45.14	11.86	0.185	11.71	0.184	7.45	0.429
Physical abuse	(−)	106.87	35.99	ref.		ref.		ref.	
	(+)	149.84	36.33	**42.97**	**0.016**	**43.35**	**0.014**	**41.56**	**0.027**
Emotional abuse	(−)	108.00	36.09	ref.		ref.		ref.	
	(+)	125.86	64.21	17.86	0.250	18.26	0.232	3.08	0.851
Sexual abuse	(−)	109.40	39.47	ref.		ref.		ref.	
	(+)	112.65	35.01	3.25	0.872	5.53	0.782	5.19	0.795

Significance at p < 0.05 are in bold.

† adjusted for age;

†† *physical neglect, emotional neglect, physical abuse, emotional abuse, sexual abuse, and age are adjusted*.

**Figure 1:**
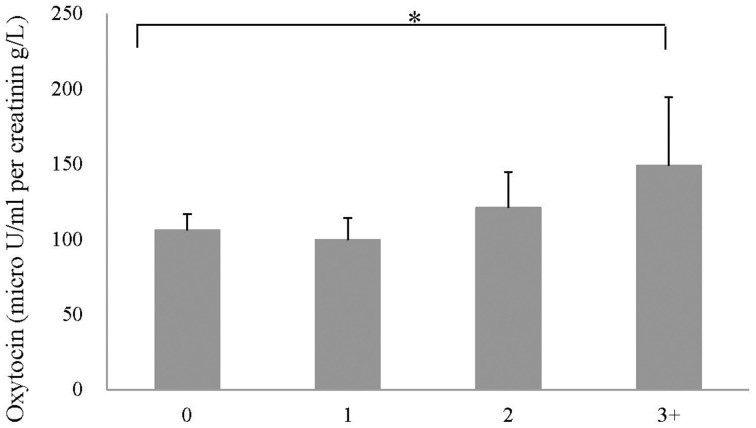
**Mean concentration for number of less severe forms of child maltreatment types**. *Note*. ^*^p = 0.013. *p* for trend = 0.031.

The association between accumulated exposure to different types of maltreatment (i.e., poly-victimization) and OT concentration was analyzed (data not shown). Participants who experienced two, or three and more, types of less severe forms of maltreatment had 12.56 and 41.91 μU/ml per creatinin g/L higher OT concentration, respectively, compared to the no-maltreatment group, and three and more types was statistically significant (*p* = 0.013). Moreover, *p* for trend was significant (*p* = 0.031), which suggested a dose-response association between the number of less severe forms of childhood maltreatment types and OT concentration (Figure 1).

The same analyses were stratified by sex. A stronger association was found among men; in the adjusted model, men who experienced three and more childhood maltreatment types showed 51.8 μU/ml per creatinin g/L higher OT concentration compared to men with no childhood maltreatment history (*p* = 0.023). The association between number of childhood maltreatment types and OT was marginal among women.

## Discussion

The current study showed that a history of less severe forms of childhood physical abuse was significantly associated with an elevated urinary OT concentration among healthy adults. A positive dose-response association was also revealed between the number of less severe forms of childhood maltreatment types and OT concentration. The current findings apply to a population sampled within Tokyo, Japan.

As OT concentration can be determined not only by childhood maltreatment, but also by other stressful events (Emeny et al., 2015), HPA reactivity (Cox et al., 2015), or inflammation (Carnio et al., 2006), the findings should be interpreted with caution. That is, we found a positive association between childhood maltreatment and OT levels, which may not be directly associated with or mediated by these unmeasured factors. Nonetheless, the finding is novel as it focuses on less severe forms of child maltreatment. This is inconsistent with past findings, which indicates inverse associations between OT concentrations and severe childhood maltreatment (Heim et al., 2009; Opacka-Juffry and Mohiyeddini, 2012), as well as an absence of the association (Wismer Fries et al., 2005). However, our results were supported by previous findings that showed social stress increased OT among people with a history of less severe forms of childhood maltreatment (Seltzer et al., 2014). Although the reasons for these inconsistent results are not clear, the current study sample is a healthy adult population with a history of less severe forms of child maltreatment in Japan. This differs from previous studies (Wismer Fries et al., 2005; Heim et al., 2009) in which samples included all adults with a history of severe forms of childhood maltreatment, such as a history of institutional care or interactions with child protective services. It has been suggested that the threat which provokes fear in humans could be divided into the categories of “threat” or “challenge” (Blascovich and Mendes, 2010). When childhood maltreatment is very severe, children may regard abusers as a “threat” which elicits a fight-or-flight response (Cannon, 1932), rather than viewing abusers as a “challenge” and attempting to resolve the situation by engaging in communication with the abuser (i.e., approach-oriented behavior). Thus, it could be speculated that exposure to severe maltreatment might reinforce the fight-or-flight response which could contribute to the down-regulation of the OT system, as described in previous studies (Wismer Fries et al., 2005; Heim et al., 2009), while less severe forms of maltreatment could foster up-regulation of the system.

Positive association between a history of less severe forms of childhood physical abuse and OT concentration could be explained by the “tend-and-befriend” response (Taylor et al., 2000). For children, running away from their abusive caregiver is accompanied by a significant risk of survival failure and is often an unrealistic option due to a child's limited resources. Thus, the OT system might be activated, which could promote approach-oriented responses and help to maintain social engagement with their abusive caregivers (Blascovich and Mendes, 2010). The reason why the OT system remains at a higher level until adulthood is unknown; however, less severe forms of childhood maltreatment might be associated with attachment style or the marital relationship (Bailey et al., 2007), which is associated with OT concentration (Samuel et al., 2015).

Emotional and sexual abuse on the other hand can affect children differently. Literature suggests that various impacts are observed depending on the type of maltreatment. Contrary to physical abuse, which is associated with aggression from the caregiver, emotional abuse is associated with the caregiver's internalized problems, such as low self-esteem (Briere and Runtz, 1990; Mullen et al., 1996), anxiety, depression, and somatization (Spertus et al., 2003). Such internalized problems may not be recognized as a threat or challenge for children to avoid, and so children may simply accept and adapt to the abuse. In such cases, it is likely that the tend-and-befriend response (Taylor et al., 2000) may not be activated and OT may not be released to the same level as with physical abuse. Similarly, children who were sexually abused might be too young to understand that their engagement in sexual activities is a form of exploitation with potential harms (i.e., threat). As CTQ does not include the age of victimization, it is not possible to assess the age of sexual abuse victimization. Unlike physical abuse, children may not recognize emotional or sexual victimization as a stressor, and hence the OT system may not be duly activated by emotional or sexual abuse.

Child neglect, defined by a failure to adequately care for a child's physical and emotional needs, signifies an absence of or a diminished amount of parental engagement in the child's life (Strathearn, 2011). Unlike physical abuse that involves violence and provokes a strong sense of fear, acts of neglect may not provoke fear in children in the same way. Without fear, the stress response system cannot be activated and actions, such as running away from abusive caregivers or engaging with them in order to procure sufficient and sensitive care, cannot occur. If the stress responses of neglected children are not activated, a positive feedback loop of OT cannot be established.

The current data showed a gradient effect of poly-victimization for less severe forms of childhood maltreatment. To the best of our knowledge, this is the first study to report a dose-response association between a history of less severe forms of childhood maltreatment and OT concentration in adulthood. This result indicates that the impact of various, less severe forms of maltreatment accumulates, and OT concentration in adulthood is enhanced. Particularly, when people have experienced three or more types of maltreatment at low or moderate levels, their OT concentration in adulthood becomes higher by 41.91 μU/ml per creatinin g/L (equivalent to 1 SD) compared to people with no history of maltreatment. It could be interpreted that a history of less severe forms of maltreatment might be related to higher OT levels regardless of maltreatment type, due to the accumulation effect of poly-victimization.

This study has several limitations. First, the sample size is small, and warrants further replication studies with larger samples, which could enable stratification by sex. Second, though urinary OT concentration as a proxy for central OT was used for its validity, other peripheral or central samples (i.e., cerebrospinal fluid) and repeated measurements are needed. Further, urinary OT concentration was measured only once. As we could not collect urine samples across several days or at different times of the day, we were unable to investigate if the fluctuation of OT concentration was due to the current family environment, such as the marital relationship. Third, the validity and reliability of retrospective self-reporting on childhood trauma is debatable due to possible underreporting and recall bias, which may lead to significant measurement errors. Since OT concentration may influence memory retrieval and self-perception, people with higher OT concentrations might have reported maltreatment history more frequently than those with lower OT concentration (Bartz et al., 2010; Cardoso et al., 2012). Further prospective studies that measure the baseline childhood maltreatment history and OT concentration via follow-up are necessary. Fourth, given the study's cross-sectional design, the current results do not indicate any causal relationship, i.e., those who showed higher OT concentrations might be more likely to recall and report a history of less severe forms of child maltreatment. Fifth, oxytocin receptor gene variations are not assessed in the current study. In addition to the level of OT secreted peripherally or centrally, further examination of the OT receptor gene and its expression is crucial to better our understanding of how the OT system functions (Nomura et al., 2003). Sixth, as all participants in the sample were married, which might be associated with both a history of child maltreatment and OT concentration (McCauley et al., 1997), this may preclude the generalizability of the findings to unmarried adults.

This study also provided several tentative implications. It could be speculated that the severity of childhood maltreatment history has an important impact on OT concentrations in adulthood. Experiences of a certain degree of social stress with parents in childhood could facilitate sensitive interactions with others and social engagement. Although the mechanism is unknown, the current study suggests the importance of measuring the severity of childhood maltreatment to interpret the OT concentration.

In conclusion, less severe forms of childhood physical abuse history were associated with higher OT concentrations among healthy adults in Japan. Poly-victimization among participants with a history of less severe forms of childhood maltreatment was also associated with a higher OT concentration. Further study is needed to elucidate the mediating factors, such as stress coping skills, for the positive association between less severe forms of childhood maltreatment and OT concentration.

### Conflict of interest statement

The authors declare that the research was conducted in the absence of any commercial or financial relationships that could be construed as a potential conflict of interest.
